# Estimated Kidney Tubular Secretion and Kidney, Cardiovascular, and Mortality Outcomes in CKD: The Systolic Blood Pressure Intervention Trial

**DOI:** 10.1016/j.xkme.2022.100546

**Published:** 2022-09-23

**Authors:** Simon B. Ascher, Michael G. Shlipak, Ronit Katz, Alexander L. Bullen, Rebecca Scherzer, Stein I. Hallan, Alfred K. Cheung, Kalani L. Raphael, Michelle M. Estrella, Vasantha K. Jotwani, Jesse C. Seegmiller, Joachim H. Ix, Pranav S. Garimella

**Affiliations:** 1 Kidney Health Research Collaborative, Department of Medicine, San Francisco Veterans Affairs Health Care System and University of California San Francisco, San Francisco, California; 2 Division of Hospital Medicine, University of California Davis, Sacramento, California; 3 Department of Obstetrics and Gynecology, University of Washington, Seattle, Washington; 4 Division of Nephrology-Hypertension, University of California San Diego, San Diego, California; 5 Nephrology Section, Veterans Affairs San Diego Healthcare System, San Diego, California; 6 Department of Clinical and Molecular Medicine, Faculty of Medicine, Norwegian University of Science and Technology, Trondheim, Norway; 7 Department of Nephrology, St Olav University Hospital, Trondheim, Norway; 8 Division of Nephrology and Hypertension, University of Utah Health, Salt Lake City, Utah; 9 Medical Service, Veterans Affairs Salt Lake City Health Care System, Salt Lake City, Utah; 10 Division of Nephrology and Hypertension, Department of Medicine, Oregon Health and Science University and VA Portland Health Care System, Portland, Oregon; 11 Department of Laboratory Medicine and Pathology, University of Minnesota, Minneapolis, Minnesota

**Keywords:** CKD progression, CVD, hypertension, kidney function decline, mortality, tubular secretion

## Abstract

**Rational & Objective:**

Many drugs, metabolites, and toxins are cleared by the kidneys via tubular secretion. Whether novel endogenous measures of tubular secretion provide information about kidney, cardiovascular, and mortality risk is uncertain.

**Study Design:**

Longitudinal subgroup analysis of clinical trial participants.

**Setting & Participants:**

2,089 Systolic Blood Pressure Intervention Trial participants with estimated glomerular filtration rate (eGFR) <60 mL/min/1.73 m^2^ at baseline.

**Exposure:**

Summary score incorporating urine-to-plasma ratios of 10 endogenous secretion markers measured in paired urine and plasma samples at baseline.

**Outcome:**

The primary outcome was longitudinal change in eGFR. Secondary outcomes included chronic kidney disease (CKD) progression (≥50% eGFR decline or incident kidney failure requiring dialysis or kidney transplantation), a cardiovascular disease (CVD) composite (myocardial infarction, acute coronary syndrome, stroke, acute decompensated heart failure, or death from cardiovascular causes), and mortality.

**Analytical Approach:**

Linear mixed-effect models were used to evaluate the association between the secretion score and change in eGFR, and Cox proportional hazards models were used to evaluate associations with CKD progression, CVD, and mortality.

**Results:**

At baseline, mean age was 73 ± 9 years and eGFR was 46 ± 11 mL/min/1.73 m^2^. During a median follow-up of 3.3 years, mean change in eGFR was −1.44% per year, and 72 CKD progression events, 272 CVD events, and 144 deaths occurred. In multivariable analyses, lower secretion score was associated with faster eGFR decline and greater risk of CKD progression, CVD, and mortality. After further adjustment for baseline eGFR and albuminuria, each 1-standard deviation lower secretion score was associated with faster eGFR decline (−0.65% per year; 95% CI, −0.84% to −0.46%), but not CKD progression (HR, 1.23; 95% CI, 0.96-1.58), CVD (HR, 1.02; 95% CI, 0.89-1.18), or mortality (HR, 0.90; 95% CI, 0.74-1.09). The secretion score association with eGFR decline appeared stronger in participants with baseline eGFR <45 mL/min/1.73 m^2^ (*P* for interaction < 0.001).

**Limitations:**

Persons with diabetes and proteinuria >1 g/d were excluded.

**Conclusions:**

Among SPRINT participants with CKD, lower estimated tubular secretion was associated with faster eGFR decline, independent of baseline eGFR and albuminuria, but not with CKD progression, CVD, or mortality.

## Visual Abstract

**Figure:**
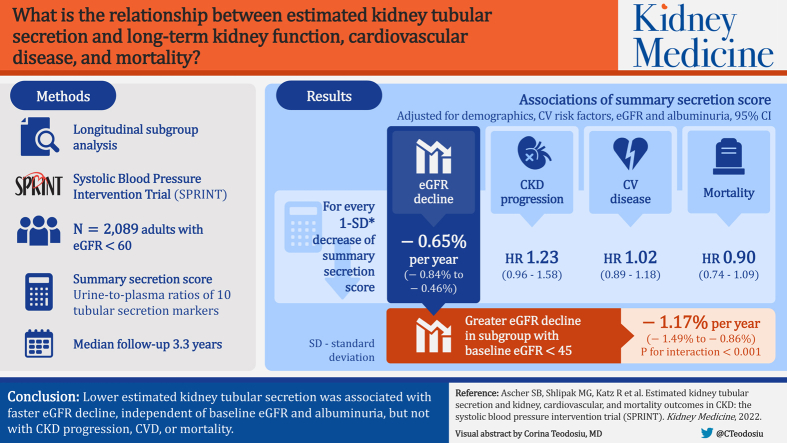

**Plain-Language Summary**

Many drugs, metabolites, and toxins are cleared by the kidneys via tubular secretion, but the clinical relevance of measuring tubular secretion has not been established. We used 10 novel endogenous markers to evaluate the association between estimated tubular secretion and risk of adverse outcomes in individuals with chronic kidney disease (CKD). Lower estimated tubular secretion was associated with faster decline in estimated glomerular filtration rate (eGFR), independent of baseline eGFR and albuminuria, but not with CKD progression, cardiovascular disease, or mortality. These findings suggest a broader assessment of kidney health that incorporates estimated tubular secretion may provide additional information about risk of kidney function decline in individuals with CKD, but contributes little additional insight about cardiovascular disease risk or survival.

Chronic kidney disease (CKD) affects nearly 10% of the global population and confers an increased risk of cardiovascular disease (CVD), kidney failure, and early death.^1^^,^^2^ Although estimated glomerular filtration rate (eGFR) and albuminuria are established risk markers for these outcomes, they primarily reflect glomerular function and injury, and do not fully capture the degree of kidney tubule pathology.^3^, ^4^, ^5^, ^6^ The kidney tubules comprise over 90% of the kidney’s cortical mass and have a central role in blood pressure regulation, electrolyte balance, and drug secretion. On kidney biopsy, interstitial fibrosis and tubular atrophy (IFTA) severity is highly prognostic of CKD progression and represents the common final pathway of nearly all forms of CKD, but these pathologic findings are poorly correlated with eGFR and albuminuria.^7^, ^8^, ^9^, ^10^ Recent studies in ambulatory populations have demonstrated that novel markers reflecting kidney tubule health, including reabsorptive capacity, injury, inflammation, and fibrosis, are associated with greater risk of longitudinal eGFR decline, acute kidney injury, CVD, and mortality.^11^, ^12^, ^13^, ^14^, ^15^ These findings suggest that a broader assessment of kidney structure and function, beyond eGFR and albuminuria, could inform prognosis in persons with CKD.^16^

Kidney tubular secretion is an essential mechanism for the clearance of many drugs, metabolites, and toxins. In contrast to glomerular filtration, secretion primarily occurs in the proximal tubule, involves extraction of protein-bound solutes directly from the peritubular capillaries, and relies on mitochondrial function for energy.^17^^,^^18^ However, the clinical relevance of measuring secretory function has not been established, in part because of a lack of standardized laboratory assays.^17^, ^18^, ^19^ Emerging evidence from the Chronic Renal Insufficiency Cohort (CRIC) Study suggests that lower estimated clearance of novel endogenous tubular secretion markers is associated with risk of CKD progression and mortality, but not CVD. These findings were independent of eGFR, albuminuria, and other CKD risk factors.^20^^,^^21^ However, the prognostic significance of tubular secretion has not been confirmed in other studies of persons with CKD.

We measured concentrations of 10 endogenous secretion markers suspected to be eliminated primarily by tubular secretion in paired plasma and urine collected at the baseline visit in Systolic Blood Pressure Intervention Trial (SPRINT) participants with eGFR <60 mL/min/1.73 m^2^. The biomarkers included: apidic acid, cinnamoylglycine, p-cresol sulfate, 1,7-dimethyluric acid, 2-furoylglycine, hippuric acid, m-hydroxy hippurate, indoxyl sulfate, phenylacetylglutamine, and tiglylglycine. These biomarkers were selected based on the following criteria: established specificity for organic anion transporters 1 and 3 (OAT1 and OAT3), which are two of the primary transporters in the proximal tubule for many common drugs, metabolites, and toxins; an increase in circulating concentrations after OAT3-transporter knockout in experimental models; a high reported protein-binding percentage; and/or kidney clearances that substantially exceed glomerular filtration rate (GFR) or creatinine clearance.^22^^,^^23^ For example, indoxyl sulfate is a gut bacteria metabolite that is highly protein-bound, undergoes elimination primarily via tubular secretion mediated by OAT1/3, accumulates in persons with CKD, and has been identified as a uremic toxin according to the European Toxin Working Group (www.uremic-toxins.org). Additional information about each secretion marker is available in the Human Metabolome Database (www.hmdb.ca). We hypothesized that baseline estimated tubular secretion, assessed by relative urine-to-plasma concentrations of the secretion markers, would be associated with faster eGFR decline and greater risk of CKD progression, CVD, and all-cause mortality, independent of baseline eGFR and albuminuria.

## Methods

### Study Design

The design and protocol of SPRINT have been reported previously.^24^^,^^25^ In brief, SPRINT was an open-label clinical trial that randomized participants with hypertension and high CVD risk to an “intensive” systolic blood pressure (BP) target of <120 mm Hg versus a “standard” BP target of <140 mm Hg. Inclusion criteria were age ≥50 years; systolic BP 130-180 mm Hg; and high CVD risk defined as prior clinical or subclinical CVD other than stroke, CKD (eGFR 20-59 mL/min/1.73 m^2^), age ≥75 years, or 10-year CVD risk >15% based on the Framingham risk score. Key exclusion criteria included diabetes mellitus, eGFR <20 mL/min/1.73 m^2^, and proteinuria > 1 g/day. A total of 9,361 participants were enrolled between November 2010 and March 2013 across 102 sites in the United States and Puerto Rico. The SPRINT protocol comprised a baseline visit and follow-up visits monthly for the first 3 months, then every 3 months thereafter. All participants provided written informed consent and the institutional review boards of all participating institutions approved the study. The trial was stopped early after a median follow-up of 3.26 years based on the recommendations of the data and safety monitoring board owing to interim CVD and mortality results that favored the intensive arm.

For this analysis, we measured 10 endogenous secretion markers in plasma and urine at baseline among 2,514 SPRINT participants with CKD, defined as a baseline eGFR <60 mL/min/1.73 m^2^ according to the 2012 Chronic Kidney Disease Epidemiology Collaboration combined creatinine and cystatin C estimating equation.^26^ We excluded 374 participants because of unavailable specimens for both plasma and urine biomarker measurements and 51 participants because of missing covariate data, resulting in a final study sample of 2,089 participants. The present study was approved by the committees on human research at the University of California, San Francisco, the San Francisco Veterans Affairs Health Care System, and the Veterans Affairs San Diego Healthcare System.

### Secretion

All paired blood and urine specimens were processed immediately, shipped overnight on dry ice, and stored at -80°C until biomarker measurement without prior thaw. Plasma and urine biomarker concentrations were measured at the SPRINT Central Laboratory (University of Minnesota, Minneapolis, Minnesota) by liquid chromatography-tandem mass spectrometry as previously described.^27^ Biomarker analytic ranges and interassay coefficients of variation are shown in Table S1. All plasma and urine solute interassay coefficients of variation were <6%. Stored urine specimens in SPRINT were from spot samples; we previously demonstrated in a pilot study that the fractional excretion of tubular secretion markers using spot urine specimens was similar to using 24-hour urine collections.^28^

We assessed tubular secretion by calculating the urine-to-plasma ratio (UPR) of each secretion marker and creating a summary score that provided a single, overall estimate of tubular secretion. We first natural log-transformed the UPR of each secretion marker, and then standardized each secretion marker to a common 0 to 100 scale based on the minimum and maximum level of log-transformed UPR of that specific marker: standardized UPR = {[ln(UPR) – min(ln[UPR])] / range(ln[UPR])} × 100, where ln(UPR) represents the natural log-transformed UPR, min(ln[UPR])] represents the minimum value in the distribution of ln(UPR), and range(ln[UPR]) represents the difference between the minimum and maximum ln(UPR) values. For each participant, we calculated the average standardized UPR ratio of the 10 secretion markers to create the summary secretion score, consistent with prior studies.^29^

### Outcomes

The primary outcome of interest was annualized eGFR slope, based on serial serum creatinine measurements collected every 3 months and measured at the SPRINT Central Laboratory. Estimated GFR was calculated by the 2009 Chronic Kidney Disease Epidemiology Collaboration equation for creatinine.^30^ Serum creatinine was measured with assays using an enzymatic creatinine method traceable to isotope dilute mass spectrometry (Roche).

The binary CKD progression endpoint was assessed as a secondary endpoint because few participants experienced this outcome in SPRINT.^24^ CKD progression was defined according to SPRINT’s primary composite kidney outcome, which required either a ≥50% eGFR decline (confirmed by repeat testing ≥90 days) or incident kidney failure requiring dialysis or kidney transplantation. We also evaluated associations of the summary secretion score with SPRINT’s primary composite CVD outcome and all-cause mortality. SPRINT’s primary composite CVD outcome included myocardial infarction, acute coronary syndrome not resulting in myocardial infarction, stroke, acute decompensated heart failure, or death from cardiovascular causes, all of which were centrally adjudicated. Clinical events occurring during follow-up were ascertained primarily through surveillance of self-reported events obtained via structured interviews every 3 months, and through laboratory and electrocardiogram data collected by the study, and were adjudicated by members of the Morbidity and Mortality subcommittee masked to treatment assignment.

### Covariates

Age, sex, race, past medical history, and smoking status were obtained by questionnaire. Trained study coordinators measured BP using a standardized protocol, and recorded BP as the mean of 3 seated BP measurements taken 1 minute apart after a 5-minute rest period using an automated oscillometric device (Model 907; Omron Healthcare).^31^ Body mass index was calculated as weight in kilograms divided by height in meters squared. Fasting serum total cholesterol, low-density lipoprotein cholesterol, high-density lipoprotein cholesterol, and triglycerides were measured at the SPRINT Central Laboratory. Urine albumin was measured by a nephelometric method using the Siemens ProSpec nephelometer (Siemens).

### Statistical analyses

Spearman correlation coefficients (*r*) were used to evaluate correlations among the secretion score, eGFR, and albuminuria. Linear mixed-effect models with random intercepts, random slopes, and an exchangeable covariance structure were used to evaluate the association of baseline secretion score with annualized eGFR slope. To interpret the slope as annualized percent change, eGFR was log-transformed. Cox proportional hazards models were used to evaluate the associations of baseline secretion score with risk of CKD progression, CVD, and all-cause mortality. We first evaluated the functional form of the secretion score association with each outcome using restricted cubic splines, adjusted for age, sex, race, and randomization arm. We modeled the secretion score both as a continuous, linear predictor (per 1-standard deviation [SD]) and categorized into quartiles. Models were sequentially adjusted for age, sex, race, intervention arm; smoking, body mass index, systolic BP, number of antihypertensive medications, history of CVD, history of heart failure, low-density lipoprotein cholesterol, high-density lipoprotein cholesterol, triglycerides, and statin use; and baseline eGFR and urine albumin-to-creatinine ratio. Participants were followed until death or the last available study visit when the trial stopped in August 2015. There was no evidence that the proportional hazards assumption was violated. Because BP targets may alter urine biomarker levels and exert hemodynamic effects on eGFR, we evaluated whether the secretion score association with each outcome varied by randomized treatment arm using a likelihood ratio test.^32^ We also evaluated whether secretion score associations varied by baseline eGFR <45 versus ≥45 mL/min/1.73 m^2^. As a sensitivity analysis, we used the Lunn-McNeil extension to the Cox model to account for the competing risk of death.^33^

All analyses were conducted using Stata (Stata Statistical Software, release 13; StataCorp LP) and SPSS (released 2016, IBM SPSS Statistics for Windows, Version 24.0, IBM Corp).

## Results

Among the 2,089 SPRINT participants with baseline eGFR <60 mL/min/1.73 m^2^ included in this analysis, mean age was 73 ± 9 years, 41% were women, and median (interquartile range [IQR]) baseline eGFR and albuminuria were 48 mL/min/1.73m ^2^ (IQR, 38-55) and 15 mg/g (IQR, 7-48), respectively. Table 1 shows the baseline characteristics stratified by the summary secretion score quartiles. Compared to participants in the lowest secretion score quartile, those in the highest quartile had higher eGFR and lower albuminuria. Baseline systolic BP and diastolic BP, the proportion randomized to each treatment arm, and the number of antihypertensive medications were similar across quartiles. Compared with SPRINT participants with baseline eGFR <60 mL/min/1.73 m^2^ not included in this analysis, those included did not have significantly different baseline characteristics (*P* ≥ 0.10 for all variables included in Table 1).

**Table 1:** Baseline Characteristics of SPRINT Participants with CKD Stratified by Summary Secretion Score Quartiles

Characteristic	Quartile 1 (N = 517)	Quartile 2 (N = 530)	Quartile 3 (N = 535)	Quartile 4 (N = 507)	All (N = 2089)
Secretion score	50 [46, 53]	58 [56, 59]	62 [61, 63]	68 [66, 70]	60 [55, 64]
Age, y	73 (10)	74 (9)	74 (9)	72 (8)	73 (9)
Female	238 (46)	197 (37)	201 (38)	218 (43)	854 (41)
Race					
Non-Hispanic White	333 (64)	356 (67)	384 (72)	328 (65)	1401 (67)
African American	137 (27)	133 (25)	108 (20)	131 (26)	509 (24)
Hispanic and other	47 (9)	41 (8)	43 (8)	48 (10)	179 (9)
BMI, kg/m^2^	29.3 (5.8)	29.3 (6.0)	29.9 (5.9)	30.0 (5.7)	29.6 (5.9)
Intensive BP arm	266 (51)	284 (54)	281 (53)	245 (48)	1076 (52)
Prevalent CVD or HF	146 (28)	173 (33)	137 (26)	132 (26)	588 (28)
Current smoker	40 (8)	55 (10)	40 (8)	45 (9)	180 (9)
eGFR, mL/min/1.73 m^2^	39 (12)	44 (10)	48 (9)	51 (7)	46 (11)
Urine ACR, mg/g	31 [10, 144]	15 [8, 56]	12 [6, 29]	10 [6, 27]	15 [7, 48]
Systolic BP, mm Hg	142 (17)	139 (16)	139 (16)	138 (17)	140 (16)
Diastolic BP, mm Hg	74 (12)	74 (12)	74 (12)	75 (13)	74 (12)
No. of antihypertensive meds	2.35 (1.07)	2.19 (1.00)	2.10 (1.00)	2.03 (0.95)	2.17 (1.01)
Total cholesterol, mg/dL	183 (41)	182 (39)	184 (41)	185 (42)	183 (41)
HDL cholesterol, mg/dL	53 (15)	52 (15)	52 (14)	52 (14)	52 (14)
Triglycerides, mg/dL	111 [79, 158]	112 [80, 149]	109 [81, 158]	112 [85, 150]	112 [82, 154]
Statin use	77 (15)	66 (13)	76 (14)	72 (14)	291 (14)

*Note*: Data displayed are mean (standard deviation), n (%), or median (interquartile range).

Abbreviations: ACR, albumin-to-creatinine ratio; BMI, body mass index; BP, blood pressure; CKD, chronic kidney disease; CVD, cardiovascular disease; eGFR, estimated glomerular filtration rate by creatinine and cystatin C; HDL, high-density lipoprotein cholesterol; HF, heart failure; SPRINT, Systolic Blood Pressure Intervention Trial.

The mean secretion score was 59.3 ± 7.6. The secretion score was moderately, positively correlated with baseline eGFR (*r* = 0.39) and inversely correlated with albuminuria (*r* = -0.30). The average UPR of each secretion marker varied significantly across baseline eGFR categories, such that the UPRs among participants with eGFR 45-59 mL/min/1.73 m^2^ were approximately 2 to 3 times higher than the UPRs among those with eGFR <30 mL/min/1.73 m^2^ (*P* < 0.001 for each tubular secretion marker, Table 2).

**Table 2:** Urine-to-Plasma Ratios of Tubular Secretion Markers Stratified by Baseline eGFR

Biomarker	eGFR, mL/min/1.73 m^2^			*P*
	45-59 (N = 1238)	30-44 (N = 641)	<30 (N = 210)	
Adipic Acid	47 [26, 76]	35 [18, 64]	22 [13, 36]	< 0.001
Cinnamoylglycine	160 [101, 239]	110 [70, 162]	67 [42, 102]	< 0.001
p-Cresol sulfate	18 [12, 26]	13 [8, 19]	7 [5, 11]	< 0.001
1,7-Dimethyluric acid	264 [172, 385]	195 [126, 295]	110 [64, 175]	< 0.001
2-Furoylglycine	406 [246, 644]	195 [177, 461]	160 [96, 277]	< 0.001
Hippuric acid	378 [248, 555]	280 [186, 415]	170 [106, 264]	< 0.001
m-Hydroxy hippurate	408 [265, 650]	307 [188, 453]	176 [102, 308]	< 0.001
Indoxyl sulfate	49 [31, 72]	34 [22, 50]	18 [11, 29]	< 0.001
Phenylacetylglutamine	268 [176, 383]	190 [128, 279]	106 [63, 169]	< 0.001
Tiglylglycine	336 [221, 484]	236 [156, 336]	137 [81, 210]	< 0.001

*Note:* Data displayed are median [interquartile range].

Abbreviation: eGFR, estimated glomerular filtration rate by creatinine and cystatin C.

**Figure 1:**
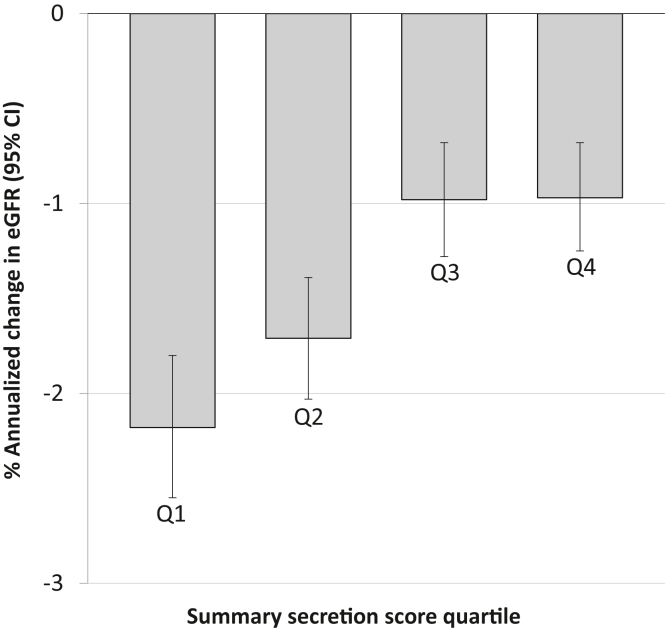
Percent annualized change in eGFR in SPRINT participants with CKD stratified by summary secretion score quartiles. Bars represent unadjusted estimated annual change in eGFR with 95% CIs displayed. Estimates are derived from linear mixed-effect models. Abbreviations: CI, confidence interval; CKD, chronic kidney disease; eGFR, estimated glomerular filtration rate; SPRINT, Systolic Blood Pressure Intervention Trial.

The mean annualized eGFR slope during the median 3.3 years of follow-up was −1.44% per year (95% confidence interval [CI], −1.60% to −1.27%). Annualized eGFR decline was fastest in the lowest secretion score quartile (Fig 1). In unadjusted analyses, lower UPRs of 9 of the 10 individual secretion markers were associated with faster eGFR decline (Table S2). After multivariable adjustment, lower UPRs of hippuric acid and m-hydroxy hippurate and higher UPR of adipic acid were associated with faster eGFR decline. Table 3 shows the association of the summary secretion score with change in eGFR. In multivariable models adjusting for demographics, clinical characteristics, intervention arm, baseline eGFR, and albuminuria, lower secretion score was significantly associated with faster annualized eGFR decline (−0.65% less per year per 1-SD lower secretion score; 95% CI, −0.84% to −0.46%). When analyzed using secretion score quartiles, the lowest quartile was significantly associated with faster eGFR decline compared with the highest quartile (−0.77% per year; 95% CI, −1.29% to −0.26%).

**Table 3:** Associations of Summary Secretion Score with Annualized eGFR Change and CKD Progression in Persons with CKD in SPRINT

**% Annualized eGFR Change**	**Mean eGFR Decline (%/y) (95% CI)**	**Model 1**^b^ **β**^a^**(95% CI)**	**Model 2**^c^ **β (95% CI)**	**Model 3**^d^ **β (95% CI)**
Per 1-SD lower score	−1.44 (−1.60 to −1.27)	−0.72 (−0.88 to −0.55)	−0.65 (−0.81 to −0.48)	−0.65 (−0.84 to −0.46)
Quartile 1^e^	−2.18 (−2.56 to −1.81)	−1.22 (−1.68 to −0.76)	−1.01 (−1.47 to −0.54)	−0.77 (−1.29 to −0.26)
Quartile 2	−1.71 (−2.03 to −1.39)	−0.64 (−1.11 to −0.18)	−0.57 (−1.03 to −0.11)	−0.39 (−0.87 to 0.08)
Quartile 3	−0.98 (−1.28 to −0.68)	0.04 (−0.41 to 0.50)	0.11 (−0.34 to 0.57)	0.04 (−0.42 to 0.49)
Quartile 4	−0.97 (−1.26 to −0.69)	Reference	Reference	Reference

**CKD progression**	**Events/N (%)**	**Model 1**^b^ **HR (95% CI)**	**Model 2**^c^ **HR (95% CI)**	**Model 3**^d^ **HR (95% CI)**
Per 1-SD lower score	72/2089 (3.4%)	1.89 (1.56-2.31)	1.87 (1.54-2.28)	1.23 (0.96-1.58)
Quartile 1	34/517 (6.6%)	4.18 (2.00-8.73)	3.99 (1.90-8.38)	0.98 (0.40-2.38)
Quartile 2	17/530 (3.2%)	1.99 (0.89-4.48)	1.84 (0.81-4.15)	0.96 (0.42-2.24)
Quartile 3	12/535 (2.2%)	1.46 (0.61-3.47)	1.45 (0.61-3.46)	1.11 (0.46-2.67)
Quartile 4	9/507 (1.8%)	Reference	Reference	Reference

Abbreviations: CI, confidence interval; CKD, chronic kidney disease; eGFR, estimated glomerular filtration rate; HR, hazard ratio; SD, standard deviation SPRINT, Systolic Blood Pressure Intervention Trial.

a β corresponds to the difference in annualized percentage change in eGFR.

b Model 1 adjusts for baseline age, sex, race, and intervention arm.

c Model 2 adjusts for Model 1 + smoking, body mass index, systolic blood pressure, number of antihypertensive medications, prevalent cardiovascular disease, low-density lipoprotein cholesterol level, high-density lipoprotein cholesterol level, triglyceride level, statin use.

d Model 3 adjusts for Model 2 + baseline eGFR and urine albumin-to-creatinine ratio.

e Median (IQR) secretion score was 60 (55-64) overall, 50 (46-53) in quartile 1, 58 (56-59) in quartile 2, 62 (61-63) in quartile 3, and 68 (66-70) in quartile 4.

**Figure 2:**
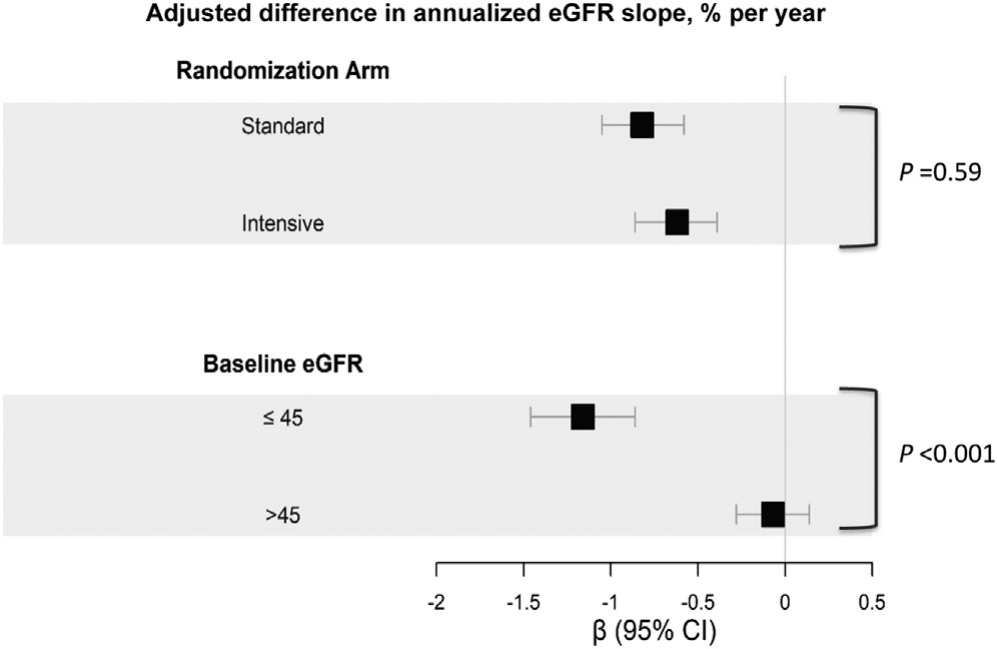
Forest plot of summary secretion score associations with difference in annualized eGFR slope in SPRINT participants with CKD stratified by intervention arm and baseline eGFR. Beta coefficients with 95% confidence intervals correspond to the difference in percent annualized eGFR slope and were obtained from linear mixed-effect models. Hazard ratios (per 1-standard deviation lower secretion score) with 95% confidence intervals obtained from multivariable Cox proportional hazards models. All models included demographics (age, sex, and race), intervention arm, clinical characteristics (smoking, body mass index, systolic blood pressure, diastolic blood pressure, number of antihypertensive medications at baseline, prevalent cardiovascular disease, low-density lipoprotein cholesterol, high-density lipoprotein cholesterol, triglycerides, and statin use), baseline eGFR, and urine albumin-to-creatinine ratio. Abbreviations: CI, confidence interval; CKD, chronic kidney disease; eGFR, estimated glomerular filtration rate; SPRINT, Systolic Blood Pressure Intervention Trial.

In subgroup analyses, lower secretion score was more strongly associated with faster eGFR decline among participants with baseline eGFR <45 mL/min/1.73 m^2^ (−1.17% per year; 95% CI, −1.49% to −0.86%), compared with participants with baseline eGFR ≥45 mL/min/1.73 m^2^ (−0.07% per year; 95% CI, −0.28% to 0.14%; *P* for interaction < 0.001; Fig 2), independent of baseline eGFR and albuminuria. Secretion score associations with eGFR decline did not vary by randomized treatment arm (*P* for interaction = 0.59, Fig 2).

The binary CKD progression endpoint occurred among 72 participants (3.4%). This endpoint occurred among 1.8% of participants in the highest secretion score quartile compared with 6.6% in the lowest quartile. In unadjusted analyses, lower UPRs of all 10 individual secretion markers were associated with greater risk of CKD progression (Table S2). After adjusting for clinical characteristics, eGFR, and albuminuria, only p-cresol sulfate and m-hydroxy hippurate remained associated with CKD progression. Lower secretion score (per 1-SD) was associated with greater risk of CKD progression in multivariable-adjusted analyses (hazard ratio [HR], 1.87; 95% CI, 1.54-2.28). However, after further adjustment for baseline eGFR and albuminuria, the association with CKD progression attenuated and was no longer statistically significant (HR, 1.23; 95% CI, 0.96-1.58). In quartile analyses, the secretion score relationship with CKD progression was relatively flat and did not reach statistical significance in fully adjusted models (Table 3).

**Figure 3:**
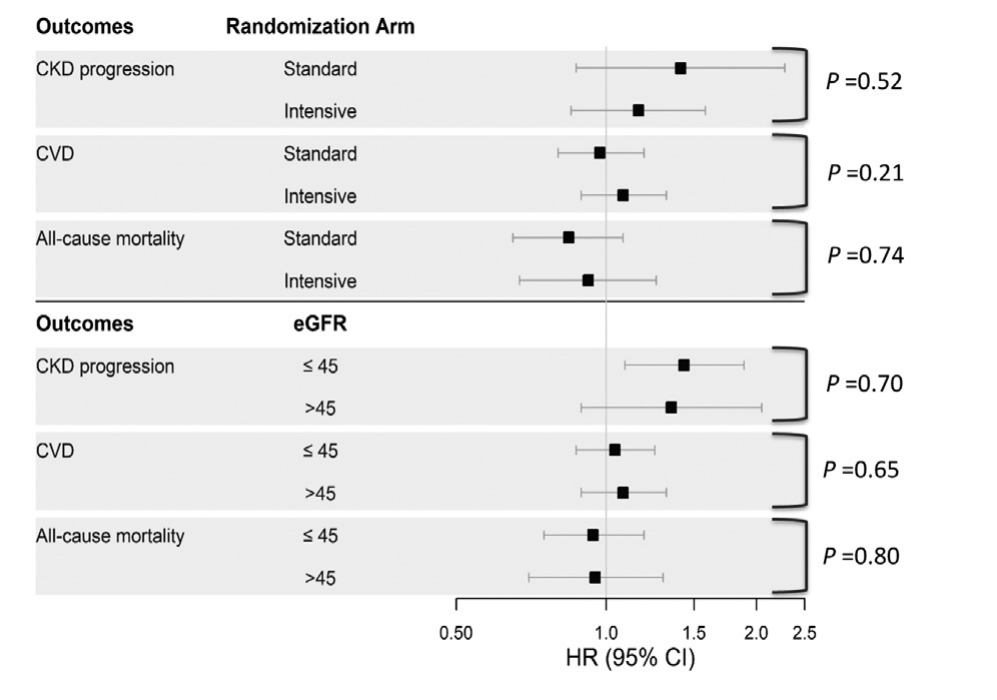
Forest plot of summary secretion score associations with risk of CKD progression, CVD, and all-cause mortality in SPRINT participants with CKD stratified by intervention arm and baseline eGFR. Hazard ratios (per 1-standard deviation lower secretion score) with 95% confidence intervals obtained from multivariable Cox proportional hazards models that included demographics (age, sex, and race), intervention arm, clinical characteristics (smoking, body mass index, systolic blood pressure, diastolic blood pressure, number of antihypertensive medications at baseline, prevalent cardiovascular disease, low-density lipoprotein cholesterol, high-density lipoprotein cholesterol, triglycerides, and statin use), baseline eGFR, and urine albumin-to-creatinine ratio. Abbreviations: CI, confidence interval; CKD, chronic kidney disease; CVD, cardiovascular disease; eGFR, estimated glomerular filtration rate; SPRINT, Systolic Blood Pressure Intervention Trial.

There were 272 participants who experienced the composite CVD endpoint (13.0%), and 144 deaths (6.9%) in the study sample. More participants in the lowest secretion score quartile experienced a composite CVD endpoint or death (16.1% and 8.9%, respectively), compared with the highest quartile (10.7% and 6.7%, respectively). In multivariable analyses, none of the individual secretion markers were associated with risk of CVD events or all-cause mortality (Table S3). In multivariable models that adjusted for demographics, clinical characteristics, and intervention arm, lower secretion score (per 1-SD) was independently associated with greater risk of the composite CVD endpoint (HR, 1.20; 95% CI, 1.07-1.36) and all-cause mortality (HR, 1.18; 95% CI, 1.00-1.39). However, after further adjustment for baseline eGFR and albuminuria, secretion score associations with both endpoints were considerably attenuated and no longer statistically significant (Table 4). Across secretion score quartiles, the relationships between secretion score and CVD and mortality were relatively flat and did not reach statistical significance in the fully adjusted model. Secretion score associations with CKD progression, CVD, and mortality did not vary by baseline eGFR <45 versus ≥45 mL/min/1.73 m^2^ or by randomized treatment arm (*P* > 0.20 for all interaction tests; Fig 3).

**Table 4:** Associations of Composite Secretion Score with CVD Events and All-Cause Mortality in Persons with CKD in SPRINT

	Events/N (%)	Model 1^a^ HR (95% CI)	Model 2^b^ HR (95% CI)	Model 3^c^ HR (95% CI)
**CVD events**				
Per 1-SD lower score	272/2089 (13.0%)	1.23 (1.09-1.39)	1.20 (1.07-1.36)	1.02 (0.89-1.18)
Quartile 1^d^	83/517 (16.1%)	1.64 (1.16-2.31)	1.57 (1.11-2.23)	1.03 (0.70-1.52)
Quartile 2	72/530 (13.6%)	1.23 (0.86-1.75)	1.17 (0.82-1.67)	0.96 (0.66-1.38)
Quartile 3	63/535 (11.8%)	1.07 (0.74-1.53)	1.07 (0.75-1.55)	1.00 (0.69-1.44)
Quartile 4	54/507 (10.7%)	Reference	Reference	Reference
**All-cause mortality**				
Per 1-SD lower score	144/2089 (6.9%)	1.22 (1.04-1.44)	1.18 (1.00-1.39)	0.90 (0.74-1.09)
Quartile 1	46/517 (8.9%)	1.42 (0.91-2.22)	1.33 (0.85-2.09)	0.66 (0.39-1.13)
Quartile 2	38/530 (7.2%)	1.03 (0.85-1.64)	0.96 (0.60-1.53)	0.68 (0.42-1.11)
Quartile 3	26/535 (4.9%)	0.71 (0.43-1.19)	0.71 (0.43-1.19)	0.63 (0.37-1.05)
Quartile 4	34/507 (6.7%)	Reference	Reference	Reference

Abbreviations: CI, confidence interval; CKD, chronic kidney disease; CVD, cardiovascular disease; HR, hazard ratio; SD, standard deviation; SPRINT, Systolic Blood Pressure Intervention Trial.

a Model 1 adjusts for baseline age, sex, race, and intervention arm.

b Model 2 adjusts for Model 1 + smoking, body mass index, systolic blood pressure, number of antihypertensive medications, prevalent cardiovascular disease, low-density lipoprotein cholesterol level, high-density lipoprotein cholesterol level, triglyceride level, statin use.

c Model 3 adjusts for Model 2 + baseline eGFR and urine albumin-to-creatinine ratio.

d Median (IQR) secretion score was 60 (55-64) overall, 50 (46-53) in quartile 1, 58 (56-59) in quartile 2, 62 (61-63) in quartile 3, and 68 (66-70) in quartile 4.

Summary secretion score associations with CVD and CKD progression did not differ when accounting for the competing risk of death using Lunn-McNeil models.

## Discussion

Tubular secretion is an essential kidney function, but its prognostic implications in persons with CKD have yet to be fully characterized. In this ancillary study of SPRINT participants with CKD at baseline, worse estimated tubular secretion was associated with faster eGFR decline independent of baseline eGFR and albuminuria. This association was stronger among the subgroup of participants with baseline eGFR <45 mL/min/1.73 m^2^. In contrast, estimated tubular secretion was not associated with risk of CVD or all-cause mortality independent of eGFR and albuminuria.

Our findings are consistent with work using novel secretion measures in the CRIC Study, Seattle Kidney Study, and the Modified Diet in Renal Disease (MDRD) Study. These studies found that worse estimated tubular secretion is associated with faster declines in eGFR longitudinally and greater risk of CKD progression, independent of baseline eGFR and albuminuria.^20^^,^^23^^,^^34^ Similar to our findings, studies in CRIC observed no association between estimated tubular secretion and CVD risk, but found that worse estimated tubular secretion was independently associated with all-cause mortality.^20^^,^^21^ In the MDRD Study, worse secretion of creatinine was associated with kidney failure risk but not CVD or all-cause mortality.^34^

An association between lower estimated tubular secretion and greater risk of CKD progression also appeared compatible with our data, although the finding did not reach statistical significance. This may have been because of insufficient power for this binary endpoint, which only occurred in 72 participants. SPRINT was designed as a CVD endpoint trial, and few CKD progression events accrued because the trial excluded individuals with baseline eGFR <20 mL/min/1.73 m^2^ or severe proteinuria, and there was a relatively short follow-up period. For this reason, we a priori selected longitudinal change in eGFR as our primary endpoint for this analysis. Collectively, these findings support and reaffirm previous findings that measurement of tubule secretion identifies individuals at higher risk of loss of kidney function, independent of eGFR and albuminuria, and that the association with loss of kidney function appears much stronger than that for CVD or all-cause mortality.

We hypothesized that measures of tubular secretion could contribute additional prognostic information about kidney outcomes, CVD, and all-cause mortality above and beyond measurements of glomerular health for several reasons. First, recent work by our group demonstrated that lower estimated tubular secretion is associated with biopsy-proven IFTA independent of eGFR and albuminuria,^29^ and prior studies consistently demonstrated that IFTA on biopsy are strongly prognostic of CKD progression.^8^^,^^10^ Second, tubular secretion occurs primarily in the proximal tubules, and novel biomarkers reflecting proximal tubule damage and dysfunction are independently associated with longitudinal eGFR decline, CKD progression, CVD, and all-cause mortality in SPRINT CKD participants.^11^^,^^12^^,^^14^^,^^15^ Third, lower tubular secretion suggests a reduced ability to clear uremic toxins, which have been implicated as a pathophysiologic driver of vascular calcification, endothelial dysfunction, inflammation, and fibrosis.^35^^,^^36^

We found that the relationship between lower estimated tubular secretion and faster eGFR decline was stronger in those with baseline eGFR < 45 mL/min/1.73 m^2^ relative to those with milder CKD. Previous studies have shown that even though estimated tubular secretion can vary considerably at any given level of GFR, average secretory function decreases in parallel with declining filtration function.^23^^,^^37^ Similarly, we observed progressively lower estimated tubular secretion across lower eGFR categories uniformly for each of the tubular secretion markers. We hypothesize that tubular secretion may become increasingly important to maintain homeostasis and carry out the kidney’s important biological functions as GFR declines. Further investigations are warranted to better understand how novel measures of tubular secretion relate to adverse outcomes at different CKD stages.

As an ancillary study of SPRINT, this analysis benefited from the inclusion of a large CKD population, frequent and protocol driven eGFR assessments during follow-up, and clinically adjudicated outcomes. We also used a broad panel of candidate secretion markers, and were able to quantify the concentrations of 10 metabolites from a single liquid chromatography-tandem mass spectrometry run. Future analyses could evaluate whether fewer markers may achieve effective characterization of estimated tubular secretory function. There are also important limitations. First, because of the SPRINT design, our findings may not generalize to persons with CKD who have severe proteinuria, eGFR <20 mL/min/1.73 m^2^, or diabetes mellitus. In addition, the summary secretion score was internally derived from tubular secretion measures from SPRINT participants. However, the distribution of summary secretion scores was similar to the distribution of scores observed in the CRIC Study using similar tubular secretion markers, suggesting the present study captures a common range of estimated tubular secretory function in CKD. In addition, our findings were similar when we evaluated the secretion markers individually. Second, because direct measurements of tubular secretion are not available, we estimated tubular secretory function using relative urine-to-plasma concentrations of endogenous secretion markers that are cleared primarily by tubular secretion.^22^^,^^23^ We previously demonstrated that spot urine-to-plasma concentrations of the secretion markers are associated with IFTA severity on kidney biopsy.^29^ Third, stored urine specimens in SPRINT were spot samples, and the secretion of individual markers may be subject to intraindividual variability.^38^ However, any misclassification due to missed variability would have biased our results toward the null. Future studies evaluating how using spot urine measurements to estimate tubular secretion compare with 24-hour urine collections are needed*.* Finally, we did not have information on certain medications such as antibiotics or antacids that may affect tubular secretion.

In summary, among persons with hypertension and CKD, lower estimated tubular secretion was associated with faster eGFR decline, independent of baseline eGFR and albuminuria. This relationship was stronger in persons with more advanced CKD at baseline. In contrast, lower estimated tubular secretion was not independently associated with a binary CKD progression endpoint, CVD composite endpoint, or all-cause mortality after adjusting for baseline eGFR and albuminuria. Overall, our findings suggest that a broader assessment of kidney health that incorporates estimates of tubular secretion may provide additional information about subsequent risk of kidney function decline but contributes little additional insight about CVD risk or survival. Additional studies are needed to validate these findings in other CKD cohorts, to determine the importance of monitoring tubular secretory capacity for predicting long-term changes in kidney function and for assessing treatment efficacy and safety, and to investigate the prognostic contributions of tubular secretion measures for other adverse outcomes in persons with CKD.

## Supplementary Material

Supplementary File (PDF)

**Table S1**: Laboratory Characteristics of Endogenous Secretory Solutes Measured in SPRINT Participants with CKD

**Table S2**: Associations of Individual Tubular Secretion Markers with Annualized eGFR Change and CKD Progression in Persons with CKD in SPRINT

**Table S3**: Associations of Individual Tubular Secretion Markers with CVD Events and All-Cause Mortality in Persons with CKD in SPRINT
