# Supplementary material for: Estimated Kidney Tubular Secretion and Kidney, Cardiovascular, and Mortality Outcomes in CKD: The Systolic Blood Pressure Intervention Trial
Source: Kidney Med. 2022 Sep 23;4(12):100546. doi: 10.1016/j.xkme.2022.100546 (PMC9732413; doi:10.1016/j.xkme.2022.100546)
Supplement: Supplementary File (PDF) [file mmc1.docx]

**Supplementary Material Table of Contents:**

**Supplementary Table S1. Laboratory characteristics of endogenous secretory solutes measured in SPRINT participants with CKD**

**Supplementary Table S2. Associations of per 1-SD lower individual tubular secretion markers with annualized eGFR change and CKD progression in persons with CKD in SPRINT**

**Supplementary Table S3. Associations of per 1-SD lower individual tubular secretion markers with CVD events and all-cause mortality in persons with CKD in SPRINT**

**Supplementary Table S1. Laboratory characteristics of endogenous secretory solutes measured in SPRINT participants with CKD**

|  | **Plasma** | | | **Urine** | | |
| --- | --- | --- | --- | --- | --- | --- |
| **Endogenous secretory solute** | **Analytic range (µM)** | **Intra-assay CV (%)** | **Inter-assay CV (%)** | **Analytic range (µM)** | **Intra-assay CV (%)** | **Inter-assay CV (%)** |
| Adipic Acid | 0.1-30 | 4.36 | 4.82 | 5-1500 | 2.75 | 3.33 |
| Cinnamoylglycine | 0.002-5 | 2.53 | 4.17 | 0.1-250 | 1.25 | 4.47 |
| p-Cresol Sulfate | 0.2-30 | 3.28 | 3.22 | 10-1500 | 2.32 | 3.55 |
| 1,7-Dimethyluric Acid | 0.1-30 | 2.63 | 5.15 | 5-1500 | 1.93 | 4.15 |
| 2-Furoylglycine | 0.02-30 | 2.46 | 3.58 | 1-1500 | 1.50 | 3.57 |
| Hippuric Acid | 0.02-30 | 3.10 | 3.64 | 5-7500 | 2.55 | 4.52 |
| m-Hydroxy Hippurate | 0.02-30 | 2.85 | 3.97 | 1-1500 | 2.13 | 3.38 |
| Indoxyl Sulfate | 0.1-30 | 2.94 | 4.82 | 5-1500 | 2.62 | 4.55 |
| Phenylacetylglutamine | 0.01-30 | 3.15 | 3.52 | 2.5-7500 | 2.10 | 4.79 |
| Tiglylglycine | 0.01-30 | 2.29 | 5.13 | 0.5-1500 | 1.82 | 3.77 |

Abbreviations: CKD, chronic kidney disease; CV, coefficient of variation; SPRINT, Systolic Blood Pressure Intervention Trial.

**Supplementary Table S2. Associations of per 1-SD lower urine-to-plasma ratios of individual tubular secretion markers with annualized eGFR change and CKD progression in persons with CKD in SPRINT**

| **Biomarker (per 1-SD lower urine-to-plasma ratio)** | **% Annualized eGFR change** | |  | **CKD progression** | |
| --- | --- | --- | --- | --- | --- |
|  | **Model 1^a^**  **β^c^ (95% CI)** | **Model 2^b^**  **β (95% CI)** |  | **Model 1**  **HR (95% CI)** | **Model 2**  **HR (95% CI)** |
| Adipic Acid | -0.06 (-0.22, 0.11) | **0.44 (0.27, 0.61)** |  | **1.49 (1.24, 1.80)** | 1.10 (0.88, 1.38) |
| Cinnamoylglycine | **-0.68 (-0.85, -0.50)** | -0.05 (-0.24, 0.14) |  | **1.42 (1.26, 1.61)** | 1.07 (0.83, 1.38) |
| p-Cresol Sulfate | **-0.72 (-0.89, -0.55)** | -0.05 (-0.24, 0.13) |  | **1.92 (1.60, 2.31)** | **1.30 (1.03, 1.65)** |
| 1,7-Dimethyluric Acid | **-0.59 (-0.75, -0.42)** | -0.13 (-0.30, 0.04) |  | **1.35 (1.20, 1.53)** | 1.17 (0.96, 1.44) |
| 2-Furoylglycine | **-0.57 (-0.74, -0.39)** | 0.08 (-0.11, 0.26) |  | **1.76 (1.48, 2.10)** | 1.23 (0.96, 1.58) |
| Hippuric Acid | **-0.86 (-1.04, -0.68)** | **-0.28 (-0.47, -0.09)** |  | **1.32 (1.18, 1.48)** | 1.07 (0.88, 1.31) |
| m-Hydroxy Hippurate | **-0.72 (-0.88, -0.55)** | **-0.19 (-0.37, -0.02)** |  | **1.78 (1.48, 2.13)** | **1.27 (1.01, 1.60)** |
| Indoxyl Sulfate | **-0.74 (-0.91, -0.57)** | -0.01 (-0.20, 0.18) |  | **1.73 (1.48, 2.02)** | 1.20 (0.95, 1.51) |
| Phenylacetylglutamine | **-0.74 (-0.91, -0.58)** | -0.12 (-0.30, 0.06) |  | **2.07 (1.70, 2.51)** | 1.33 (1.05, 1.70) |
| Tiglylglycine | **-0.71 (-0.87, -0.54)** | -0.07 (-0.26, 0.11) |  | **1.81 (1.51, 2.18)** | 1.17 (0.92, 1.48) |

Abbreviations: CKD, chronic kidney disease; SD, standard deviation; HR, hazard ratio; SPRINT, Systolic Blood Pressure Intervention Trial.

^a^ Model 1 adjusts for baseline age, sex, race, and intervention arm.

^b^ Model 2 adjusts for Model 1 + smoking, body mass index, systolic blood pressure, number of antihypertensive medications, prevalent cardiovascular disease, low-density lipoprotein cholesterol level, high-density lipoprotein cholesterol level, triglyceride level, statin use, baseline estimated glomerular filtration, and urine albumin-to-creatinine ratio.

^c^ β corresponds to the difference in annualized percentage change in eGFR.

**Supplementary Table S3. Associations of per 1-SD lower urine-to-plasma ratios of individual tubular secretion markers with CVD events and all-cause mortality in persons with CKD in SPRINT**

| **Biomarker (per 1-SD lower urine-to-plasma ratio)** | **CVD events** | |  | **All-cause mortality** | |
| --- | --- | --- | --- | --- | --- |
|  | **Model 1^a^**  **HR (95% CI)** | **Model 2^b^**  **HR (95% CI)** |  | **Model 1**  **HR (95% CI)** | **Model 2**  **HR (95% CI)** |
| Adipic Acid | **1.14 (1.02, 1.28)** | 1.00 (0.89, 1.14) |  | 1.11 (0.94, 1.30) | 0.89 (0.75, 1.07) |
| Cinnamoylglycine | **1.20 (1.08, 1.34)** | 1.06 (0.93, 1.20) |  | **1.15 (1.01, 1.30)** | 0.91 (0.76, 1.10) |
| p-Cresol Sulfate | **1.24 (1.10, 1.39)** | 1.02 (0.89, 1.16) |  | **1.28 (1.09, 1.49)** | 0.96 (0.80, 1.14) |
| 1,7-Dimethyluric Acid | 1.09 (0.98, 1.21) | 0.96 (0.84, 1.09) |  | 1.10 (0.95, 1.27) | 0.87 (0.72, 1.06) |
| 2-Furoylglycine | **1.14 (1.01, 1.28)** | 0.96 (0.85, 1.09) |  | 1.08 (0.92, 1.26) | 0.82 (0.70, 0.97) |
| Hippuric Acid | **1.11 (1.01, 1.22)** | 0.99 (0.87, 1.12) |  | 1.11 (0.97, 1.27) | 0.90 (0.74, 1.09) |
| m-Hydroxy Hippurate | **1.26 (1.12, 1.41)** | 1.11 (0.98, 1.26) |  | **1.20 (1.02, 1.41)** | 0.96 (0.80, 1.15) |
| Indoxyl Sulfate | **1.25 (1.12, 1.39)** | 1.02 (0.89, 1.17) |  | **1.28 (1.11, 1.48)** | 0.95 (0.78, 1.14) |
| Phenylacetylglutamine | **1.24 (1.10, 1.39)** | 1.01 (0.88, 1.15) |  | **1.20 (1.02, 1.41)** | 0.86 (0.71, 1.03) |
| Tiglylglycine | **1.24 (1.10, 1.39)** | 1.04 (0.91, 1.18) |  | **1.25 (1.07, 1.46)** | 0.93 (0.77, 1.12) |

Abbreviations: CVD, cardiovascular disease; SD, standard deviation; HR, hazard ratio; SPRINT, Systolic Blood Pressure Intervention Trial.

^a^ Model 1 adjusts for baseline age, sex, race, and intervention arm.

^b^ Model 2 adjusts for Model 1 + smoking, body mass index, systolic blood pressure, number of antihypertensive medications, prevalent cardiovascular disease, low-density lipoprotein cholesterol level, high-density lipoprotein cholesterol level, triglyceride level, statin use, baseline estimated glomerular filtration, and urine albumin-to-creatinine ratio.
